# Molecular Testing in Stage 4 Stomach Cancer in India: A Single-Centre Experience

**DOI:** 10.7759/cureus.49412

**Published:** 2023-11-25

**Authors:** Rahul Anand, Amit Rauthan, Poonam Patil, Nitin Y Murthy

**Affiliations:** 1 Medical Oncology, Manipal Hospital, Old Airport Road, Bengaluru, IND

**Keywords:** molecular markers, pdl-1, mmr, gastric cancer, her2 neu

## Abstract

Introduction

Advanced gastric cancer (GC) has a very poor prognosis, and chemotherapy has been the standard of care. The use of immunotherapy or targeted therapy in the stage 4 setting is dependent on molecular testing of the tumour. There is a paucity of data in the Indian scenario on testing for molecular markers in stage 4 GC. Therefore, in this study, we looked at the prevalence of human epidermal growth factor receptor 2 (HER2/neu) expression/amplification, deficient mismatch repair (d-MMR)/microsatellite instability (MSI) high status, and programmed death ligand 1 (PDL-1) status in stage 4 gastric/gastroesophageal junction (GEJ) adenocarcinoma.

Methods

A retrospective single-centre observational study was conducted between January 2017 and January 2022 of patients diagnosed with stage 4 GC/GEJ adenocarcinoma. Patient data were collected from stored electronic patient records. Data on stage 4 patients who underwent testing for HER2/neu, mismatch repair (MMR)/MSI, and PDL-1 status were recorded. Treatment received was also noted.

Results

During the study period, 139 patients were diagnosed with stage 4 GC/GEJ adenocarcinoma. HER2/neu testing was done in 99 stage 4 patients (71.2%), with a positivity rate of 16.16% (n = 16). All patients diagnosed as HER2/neu-positive were treated with trastuzumab. Testing of MMR status was carried out in 91 stage 4 patients (65.4%). d-MMR/MSI high was detected in eight patients (8.8%), of which germline MMR was detected as positive in one patient. Five of these eight patients (62.5%) received immune checkpoint inhibitors. PDL-1 testing was done in 61 of the 139 stage 4 patients (43.9%). Twenty patients (32.7%) had PDL-1 tumour proportion score > 1%/combined positive score > 1.

Conclusion

Molecular profiling has now become the standard while treating late-stage GC. HER2/neu-positive patients have improved survival due to the use of anti-HER2/neu-targeted therapies. It is important to look at not only PDL-1 but also MMR to identify patients who would be eligible and benefit from immunotherapy.

## Introduction

Globally, one of the leading sites of cancer incidence and cancer-related deaths is gastric cancer (GC) with 1,089,103 new cases and 768,793 deaths in 2020 [1]. GC is also the fifth most common cancer in India and the third most common cancer in Indian men as per GLOBOCAN 2020 estimates [2]. The report of the National Cancer Registry Programme, India reported that about 75% of stomach cancer patients are detected in the advanced stages (locoregional or with metastasis) and estimates over 50,000 new cases of GC annually in India by 2025 [3]. An evolution in the chemotherapy regimens for treating localised and metastatic disease and the advent of targeted therapy and immunotherapy in the metastatic setting have improved survival outcomes. The use of immunotherapy or targeted therapy in the stage 4 setting is dependent on molecular testing of the tumour.

HER2/neu (human epidermal growth factor receptor 2) receptor is a tyrosine kinase receptor, the gene for which is encoded on the 17q chromosome. It is associated with multiple cell signalling pathways involved in cell proliferation and was discovered to play an important role in tumour cell proliferation in breast cancer conferring a worse prognosis and decreased survival [4]. Blockade of this cell signalling pathway with trastuzumab improved survival in metastatic breast cancer and reduced the risk of death/recurrence in an adjuvant setting [5]. It has been widely cited that the overexpression of HER2/neu protein in GC, using immunohistochemistry (IHC), was first described in 1986 [6]. Since then, literature abounds with various studies reporting varying levels of HER2/neu positivity in GC cancer/gastroesophageal junction (GEJ) cancers with studies done in the decade of 2000s reporting a positivity across all stages to be between 13% and 23% based on the determination of HER2/neu overexpression by IHC [7]. Indian studies done on smaller sample sizes of less than 100 patients have shown a prevalence of HER2/neu-positive in GC across all stages to be between 26.92% and 56% [8-10]. HER2/neu has now been established to be a negative prognostic factor in GC with poorer survival [11]. However, with the approval of trastuzumab for the treatment of metastatic/unresectable GC/GEJ tumours and the recent approval of antibody-drug conjugates like trastuzumab deruxtecan, there exist multiple therapeutic options associated with improved efficacy for the treatment of HER2/neu-positive advanced GC tumours [12,13].

Another molecular marker of importance in GC is the mismatch repair (MMR) system wherein a deficiency in the repair system can lead to errors in repair in the microsatellite regions leading to the accumulation of mutations termed microsatellite instability (MSI) [14]. The Cancer Genome Atlas (TCGA) has characterised MSI tumours as a distinct biological subtype [15]. It is also now therapeutically imperative to identify the status of expression of the MMR proteins/MSI as studies have shown that MMR deficient (d-MMR)/MSI-high (MSI-h) tumours demonstrate good response and prolonged survival on treatment with checkpoint inhibitors such as pembrolizumab and nivolumab [16,17].

Programmed death ligand 1 (PDL-1) is a cell surface (transmembrane) protein that interacts with T-cell receptors (PD-1 receptor) and promotes tumour immune escape [18]. Targeting tumours using this axis through checkpoint inhibitors has resulted in superior outcomes in multiple tumours [19]. This is now seen even in advanced stage 4 GC where a combination of immunotherapy with chemotherapy has resulted in better outcomes as seen in the landmark Checkmate-649 trial. Though the benefit was seen across subgroups for the combination of nivolumab with chemotherapy, the magnitude of benefit was higher in those with PDL-1 combined positive score (CPS) > 5 [20].

There is a paucity of data in the Indian scenario on testing for molecular markers in stage 4 GC. Therefore, in this study, we looked at the prevalence of HER2/neu expression/amplification, d-MMR/MSI status and PDL-1 positivity in stage 4 GC/GEJ cancers.

## Materials and methods

A retrospective, single-centre observational study was conducted on patients who were diagnosed with stage-four GC/GEJ adenocarcinoma between January 2017 and January 2022. Patient data were collected from stored electronic patient records.

Demographic details, histopathology, and stage were noted. Information about HER2/neu testing and evaluation of the MMR/MSI status and PDL-1 status of those found to be stage 4 as per the 8th edition of the American Joint Committee on Cancer Staging Manual was recorded. Treatment received was also noted.

Records that showed HER2/neu IHC 3+ or IHC 2+ with subsequent fluorescence in situ hybridization (FISH) testing showing HER2/neu amplification were recorded as HER2/neu-positive cases. Those found to have reports showing a deficiency in MLH1 (MutL homolog 1)/PMS2 (PMS1 homolog 2, mismatch repair system component)/MSH2 (MutS homolog 2)/MSH6 (MutS homolog 6) by IHC or having a polymerase chain reaction (PCR) report of MSI-high were considered d-MMR/MSI-h. PDL-1 reports of tumour proportion score (TPS)/CPS were noted.

## Results

A total of 139 patients were diagnosed with stage 4 GC/GEJ adenocarcinoma between January 2017 and January 2022. The median age of the study population was 58 (IQR 47 - 63) years. Males constituted 67.6% (n = 94) of the study population. Signet ring cells were seen in 31.6% (n = 44) on histological examination. Molecular testing was performed as described in Table 1.

**Table 1:** Molecular tests performed HER2/neu: human epidermal growth factor receptor 2; MMR: mismatch repair; MSI: microsatellite instability; PDL-1: programmed cell death ligand 1.

Molecular testing in stage 4 patients (n = 139)	Total tested	Percentage
HER2/neu testing	99	71.2%
MMR/MSI	91	65.4%
PDL-1	61	43.9%

HER2/neu testing was done in 99 stage 4 patients (71.2%) with a positivity rate of 16.16% (n = 16). Of the 16 positive cases, 11 tested positive by IHC (IHC 3+). Fourteen were IHC 2+. These were tested subsequently by FISH, and five were FISH-positive.

Of the 16 HER2-neu-positive cases, 56.25% (n = 9) were distal tumours, 31.25% (n = 5) were proximal tumours, and 12.5% (n = 2) were in the body of the stomach. As a result of testing positive for HER2/neu, all 16 patients received targeted therapy with trastuzumab.

Testing of MMR/MSI status was carried out in 91 stage 4 patients (65.4%). d-MMR/MSI high was detected in eight subjects (8.8%), of which five subjects were treated with immunotherapy with immune checkpoint inhibitors (ICI). The details of MMR-deficient proteins/MSI-high status of these patients are given in Table 2. Amongst the eight patients with d-MMR/MSI-h phenotype, testing for germline MMR mutation (suggestive of a diagnosis of Lynch syndrome) was positive in one patient.

**Table 2:** HER2/neu expression/amplification and MMR-deficient proteins/MSI-high status HER2/neu: human epidermal growth factor receptor 2; IHC: immunohistochemistry; FISH: fluorescence in situ hybridization; d-MMR: deficient mismatch repair; MSI-h: microsatellite instability-high; MLH1: MutL homolog 1; PMS2: PMS1 homolog 2.

Molecular marker		Total population tested (n)	Frequency		Percentage (% of n)	
HER2/neu-positive (IHC 3+/IHC 2+ & FISH+)		99	16		16.16	
IHC 3+			11		11.11	
IHC 2+			14		14.14	
IHC 2+ and FISH+	IHC2+ and FISH-		5	9	5.05	9.05
d-MMR/MSI-h status		91	8		8.8	
PMS2 deficient			1		1.1	
MLH1 deficient			1		1.1	
PMS2 and MLH1 deficient			3		3.3	
MSI high			3		3.3	

PDL-1 testing was done in 61 of the 139 stage 4 patients (43.9%). CPS/TPS < 1 was recorded as negative. Twenty (32.7%) tested PDL-1 TPS > 1%/CPS > 1, and 41 (67.2%) tested negative for PDL-1, as described in Table 3.

**Table 3:** PDL-1 testing results PDL-1: programmed cell death ligand 1; TPS: tumour proportion score; CPS: combined positive score.

TPS/CPS (n = 61)	Frequency	Percentage (% of n)
PDL-1 >1%	20	32.7
TPS 1-5%	16	26.2
TPS 6-10%	2	3.3
TPS 11-25%	0	0
TPS >25%	1	1.6
CPS >1	1	1.6
PDL-1 <1%	41	67.2
TPS <1%	31	50.8
CPS <1	10	16.4

A majority of patients (n = 50, 81.96%) had PDL-1 scores reported as TPS. Nineteen of these were TPS > 1%. Toward the later part of the study period, with the introduction of CPS as the standard of reporting PDL-1 in GC, 11 (18.03%) patients had a PDL-1 report of CPS out of which one had CPS > 1. Seven (35%) out of the 20 with PDL-1 score of TPS > 1%/CPS > 1 received ICI as part of their treatment.

## Discussion

Treatment of stage 4 stomach cancer has evolved with the advent of targeted therapy and immunotherapy. However, these drugs need testing for biomarkers to guide therapeutics. In this study, we report a single-centre experience of testing such biomarkers in stage 4 stomach cancer.

In concurrence with epidemiological reports, males were the predominant group in our study population [1,3]. Signet ring cells were seen in 34.2% of our patients, which has been shown to be a prognostic factor of poor outcome [21].

A HER2/neu positivity rate of 16.16% among stage 4 patients was found in our study. This is within the range of earlier studies published worldwide showing positivity rates across all stages to be between 13% and 23% [7], though lesser than the overall positivity of 22.1% in the global screening program of the landmark ToGA trial [12]. While Indian studies have reported higher HER2/neu positivity rates between 26.92% and 56% in various studies across stages, the proportion of stage 4 patients in these studies was limited [8-10].

Contrary to the majority of literature, we found a predominance of HER2/neu in distal gastric tumours as opposed to proximal/GEJ tumours [22,23]. This could be attributed to our smaller sample size and warrants further study and analysis.

Our study shows the occurrence of HER2/neu IHC 3+ and 2+ of 11.11% and 14.14%, respectively. Similar results have been obtained in studies carried out in Asian countries like China, wherein in a study carried out by Shan et al. amongst 1463 samples of patients across different stages, 9.8% had HER2/neu IHC 3+ while 14% had IHC 2+ [22].

In our study, out of the 14 patients who had IHC 2+, five out of the 14 (35.7%) demonstrated HER2/neu amplification by FISH. A higher FISH positivity of 54.6% was noted amongst the IHC2+ in the screening for the TOGA trial [12]. Identifying HER2/neu positivity had treatment implications as all patients in our centre who were diagnosed to be HER2/neu-positive went on to receive anti-HER2/neu therapy along with chemotherapy.

Identifying a d-MMR/MSI-h subtype has prognostic and therapeutic implications in GC. In our study, we report a d-MMR/MSI-h rate of 8.8% among stage 4 patients. Guan et al. in 2021 reported a d-MMR/MSI-h rate of 6.6% across all stages of GC [24], while the Asian Cancer Research Group (ACRG) reported 11.68% of patients in stage 4 to be MSI [25]. While TCGA of GC reported a higher MSI-h subgroup of 22%, the proportion of stage 4 patients was lower and the etiopathogenesis of GC between Eastern and Western countries can be different [15]. While literature is limited with regard to germline MMR mutations in gastric cancer, the available evidence points out that a majority of d-MMR/MSI-h GC phenotype occurs in a sporadic form rather than with familial clustering/germline mutations [26]. This trend was seen in our study too where only one patient amongst the eight within the d-MMR/MSI-h subgroup had a germline mutation in the MMR gene. Even amongst early-onset GC in those less than 50 years old, Bacani et al. found two germline MMR mutations out of the seven MSI-h patients giving rise to a prevalence of hereditary GC of approximately 1% among the 139 patients in their study [27].

Multiple studies looking at PDL-1 positivity with varying cut-offs of PDL-1 expression in tumour cells across stages in GC have reported positivity rates ranging from 14.32% to 69.4% [28]. A study from Japan, with a cut-off of 1%, reported PDL-1 positivity at 22.8% in tumour cells [29]. We report an occurrence of PDL-1 >1% of 32.7%. During the initial years of the study period, CPS testing had not yet become the standard for PDL-1 reporting and hence a majority of patients during the study period had PDL-1 positivity reported in terms of TPS. Later, as CPS became recognised as a standard for the reporting of PDL-1 status [20,30], our hospital moved to CPS testing. This accounts for the mix of TPS and CPS in our data.

One of the limitations of this study was that in the initial years of the study period, not all patients diagnosed with stage 4 stomach cancer had access to uniform molecular testing due to resource constraints. Furthermore, there was heterogeneity in PDL-1 testing in the initial years of the study period till CPS became the standard. This study does not look at the prognosis and survival implications of these biomarkers and further long-term follow-up studies will need to be done to evaluate these outcomes.

## Conclusions

Molecular profiling has now become the standard while treating advanced-stage GC. Our study in a real-world setting in India found around 16% of stage 4 GCs to be HER2/neu-positive. Survival of these patients has improved with the use of trastuzumab and could further improve with the use of trastuzumab deruxtecan in the future. MMR/MSI testing has become very important, as this subgroup has shown a very good response to immunotherapy and has a better prognosis, and our study showed 8% of stage 4 GCs to be MMR-deficient/MSI high. PDL-1 testing is important for decision-making for the use of immunotherapy with combination chemotherapy in the first-line setting, though this would need standardisation using CPS reporting. Management of advanced-stage GC in the present era should include HER2/neu, MMR/MSI, and PDL-1 testing, as this has a significant impact on treatment and prognosis.
